# Establishment of Reference Intervals for Serum Thyroid-related Hormones in the Chinese Oldest-old

**DOI:** 10.7150/ijms.109606

**Published:** 2025-04-28

**Authors:** Dizhi Liu, Sihang Fang, Danni Gao, Zhaoping Wang, Tenger Wang, Lei Tang, Mingjun Jiang, Juan Jiao, Hongye Zhao, Huabin Su, Rongqiao Li, Bin Huang, Yuan Lv, Guofang Pang, Caiyou Hu, Ze Yang, Huiping Yuan

**Affiliations:** 1 The Key Laboratory of Geriatrics, Beijing Institute of Geriatrics, Institute of Geriatric Medicine, Chinese Academy of Medical Sciences, Beijing Hospital/National Center of Gerontology of National Health Commission, Beijing, 100730, P.R. China.; 2 Respiratory Department, Beijing Children's Hospital, Capital Medical University, China National Clinical Research Center of Respiratory Diseases, National Center for Children's Health, Beijing, 100045, P.R. China.; 3 Department of Clinical Laboratory, the Seventh Medical Center of PLA General Hospital, Beijing, 100700, P.R. China.; 4 Department of Biochemistry and Molecular Biology, The Key Laboratory of Neural and Vascular Biology, Ministry of Education of China, Hebei Medical University, Shijiazhuang, Hebei 050017, P.R. China.; 5 Jiangbin Hospital, Guangxi Zhuang Autonomous Region, 530021, P.R. China

**Keywords:** Reference interval, Thyroid hormone, Thyroid-stimulating hormone (TSH), Thyroid function, Oldest-old

## Abstract

**Background:** Thyroid function differs between the oldest-old and younger adults, however, there is a lack of appropriate RIs for the serum FT4, T4, FT3, T3, and TSH concentrations for the oldest-old.

**Methods:** A total of 349 people was selected from the natural longevity cohort of Guangxi residents over 90 years old. The distributions of serum FT4, T4, FT3, T3, and TSH concentrations and the FT3/FT4 ratio were also analyzed. Regression curves and centile curves were constructed. Correlations between body indices and blood pressure and serum thyroid-related hormone levels were analyzed. The RIs were determined using a nonparametric approach (2.5th-97.5th percentiles) following the CLSI guidelines.

**Results:** The RIs for Chinese oldest-old are different from the current standard RIs for younger adults. A notable correlation was found between FT4 levels and age. BMI and WHtR were positively correlated with FT4, FT3, T3 and the FT3/FT4 ratio. The T3 level is correlated with SBP and DBP, and the FT3/FT4 ratio is correlated with SBP. The RIs established for the healthy oldest-old were as follows: T4, 81-193 nmol/L; FT4, 10.39-20.46 pmol/L; T3, 0.94-2.05 nmol/L; FT3, 3.56-6.43 pmol/L; TSH, 0.29-5.28 μIU/ml; and FT3/FT4, 0.197-0.496.

**Conclusions:** In this study, we established RIs of thyroid-related hormone levels for the oldest-old in China and evaluated the associations between thyroid hormone levels and body indices. These findings may provide evidence for the diagnosis and treatment of thyroid-related diseases in the oldest-old.

## Introduction

The number of older individuals worldwide has grown quickly in recent decades, and it is predicted that by the next decade, about one-third of the population in developed countries will be over 65 years old 1. In China, life expectancy has steadily increased, and the population is aging at a rapid rate 2. Aging is characterized by a gradual decline in function and increased vulnerability to disease and death, which impose major challenges in terms of elderly care and public health 3. Therefore, more attention needs to be devoted to elderly people, especially the long-lived population, in order to promote healthy aging.

The thyroid gland is one of the most important organs for secreting hormones that regulate the metabolism of proteins, fats, and glucose; influence the growth and development of the body; and increase the excitability of the central nervous system. Previous studies on thyroid-mediated regulation have focused mainly on thyroid diseases and dysfunctions. In the last few decades, thyroid hormones have been related to aging, longevity, and the occurrence of age-associated diseases, such as cardiovascular disease and diabetes 4, 5. Emerging evidence suggests that thyroid hormone levels in healthy long-lived people are different from those in the general population 6. However, the effects of differences in thyroid status on longevity remain unclear.

The main hormones produced by the thyroid gland are thyroxine (T4) and triiodothyronine (T3). Approximately 40% of secreted T4 can be deiodinated to yield T3 by enzymes in peripheral tissue. In the serum, T4 and T3 are primarily bound in a reversible manner to carrier proteins like thyroxine-binding globulin (TBG), thyroxine-binding prealbumin (TBPA), and albumin. Only small portions of T4 and T3 are known to dissociate: these portions are known as free thyroxine (FT4) and free triiodothyronine (FT3)^7^. FT4 is the primary secretory product of the functioning thyroid gland, reflecting its function and condition. In thyroidal and peripheral tissues, FT4 is converted to the active FT3 hormone, a process that can be assessed by the circulating FT3/FT4 ratio. Studies have demonstrated that the FT3/FT4 ratio is useful for differentiating various types of thyroid diseases, such as differentiating Graves' disease from destructive thyroiditis 8. In addition, the adenohypophysis releases thyroid-stimulating hormone (TSH), which regulates hormone secretion from the thyroid gland and is one of the most precise markers for assessing thyroid function 9.

The serum levels of FT4, T4, FT3, T3 and TSH, which are also known as thyroid function tests, are useful in diagnosing thyroid conditions like hyperthyroidism and hypothyroidism. Subclinical hypothyroidism is characterized by elevated serum TSH levels, while subclinical hyperthyroidism is marked by reduced serum TSH levels, both with normal serum FT4 levels 9. Thyroid diseases are among the most common disorders in China. In a survey of 78,470 participants from 31 cities in China, 50.96% were found to have thyroid conditions, with a rising trend in the elderly 10. In older adults, the diagnosis and treatment of thyroid diseases are different from those in younger individuals due to changes in the hypothalamic-pituitary-thyroid (HPT) axis with age 11.

Thyroid function tests are common assessments in clinical chemistry and are used to aid the diagnosis and guide the treatment of thyroid diseases and dysfunction. Reference intervals (RIs) are utilized to evaluate whether the test results are at expected levels. However, RIs often generate confusing conclusions since they are based on populations with different ethnographic characteristics or inappropriate samples including individuals with diagnosed or undiagnosed thyroid dysfunction, or various analysis systems. The clinical indices and biochemical indices of elderly individuals are usually different from those of younger adults. Previous studies revealed that a variety of biochemical indices change significantly with age, such as fasting plasma glucose, blood pressure, blood lipids and C-reactive protein 12. Serum thyroid hormones and TSH levels can be affected by age, ethnicity, sex, iodine intake, etc. The National Academy of Clinical Biochemistry suggested that the RIs of serum thyroid function-related hormones should be established for euthyroid individuals after rigorous screening 13. RIs derived from young people are widely used domestically and internationally, but specific RIs for the oldest-old are unavailable worldwide 14. Therefore, developing standardized RIs for the Chinese oldest-old is highly important for improving the clinical management of the elderly thyroid diseases and for promoting healthy aging.

However, it remains unclear whether the serum FT4, T4, FT3, T3, and TSH concentrations and the FT3/FT4 ratio differ between younger adults and the oldest-old and whether these parameters change with age. In this study, we explored the relationships between age, sex, body-related indices, blood pressure and thyroid-related hormones. Moreover, we established RIs for FT4, T4, FT3, T3, and TSH concentrations and the FT3/FT4 ratio for local oldest-old in Guangxi, China.

## Materials and methods

### Subjects

The study was conducted using data from a natural longevity cohort in Guangxi, China. Only apparently healthy individuals were included as potential reference individuals according to the Clinical and Laboratory Standards Institute (CLSI) guidelines 15. The exclusion criteria were as follows: (1) bedridden participants; (2) subjects with past medical history of thyroid diseases; (3) individuals applying thyroid-related medications; and (4) subjects with other serious or acute diseases.

People who are more than 90 years old were defined as the oldest-old 16. After screening the original subjects and eliminating data missing individuals, a total of 349 individuals were included in the present study, with ages ranging from 90-108 years. There were 139 men (39.8%) and 210 women (60.2%) included in this study.

### Anthropometric and biochemical measurements

Height and weight measurements were taken using a standardized tool. BMI was calculated as bodyweight (kg) divided by squared body height (m^2^). Waist and hip circumferences were measured using a nonelastic plastic measuring tape from the midpoint to the nearest 0.1 cm. The waist-hip ratio (WHR) was defined as the waist circumference (cm) divided by the hip circumference (cm). The waist height ratio (WHtR) was defined as the waist circumference (cm) divided by the height (cm). Blood pressure was measured twice after the participants had rested for at least 10 minutes using a mercury-column sphygmomanometer. Subsequently, the means for both systolic and diastolic blood pressure were calculated.

All participants fasted for at least 8 hours before blood was collected. Blood samples were obtained with anticoagulant tubes containing 1% EDTA and preserved at -80 °C. Jiangbin Hospital in Guangxi Province was responsible for the storage and analysis of all the specimens. Serum FT4, T4, FT3, T3 and TSH concentrations were measured according to standard laboratory procedures using the Roche Cobas system 17. FT3 (pmol/L) divided by FT4 (pmol/L) was used to calculate the FT3/FT4 ratio. The measurements were conducted in strict accordance with the standard operating procedure.

### Statistical analysis

All the data were analyzed by SPSS Statistics version 21.0 (IBM Corporation, Armonk, NY). A normal distribution was evaluated with the Kolmogorov-Smirnov test. Continuous variables that are normally distributed are expressed as means ± standard deviations (SD). Spearman's correlation coefficients were calculated as appropriate to determine the association between thyroid hormone levels and sex. The correlations between age, body-related indices (BMI, WHR, WHtR), and blood pressure (SBP, DBP) were evaluated using Pearson's correlation analysis. p < 0.05 was considered to indicate statistical significance.

Chinese standard RIs for FT4, T4, FT3, T3, and TSH concentrations and the FT3/FT4 ratio were based on the reference intervals for common clinical biochemistry tests (WS/T 404.10—2022) published by the National Health Commission of the People's Republic of China 18. An outlier was defined as a data point outside the lower limit or upper limit of Chinese standard RIs.

The Generalized Additive Models for Location, Scale and Space (GAMLSS) model was used to plot the centile curves for the distributions of FT4, T4, FT3, T3, TSH and FT3/FT4. The GAMLSS algorithm was implemented using the R3.6.3 software GAMLSS package (version 4.3-3) by Rigby and Stasinopoulos 19. Finally, a nonparametric method was used to set up the reference intervals according to the authoritative guidelines of the CLSI 15. RIs are defined as the interval between two limiting values: the 2.5% position was used as the lower limit, while the 97.5% position was used as the upper limit.

## Results

### Characteristics of the subjects

**Figure 1:**
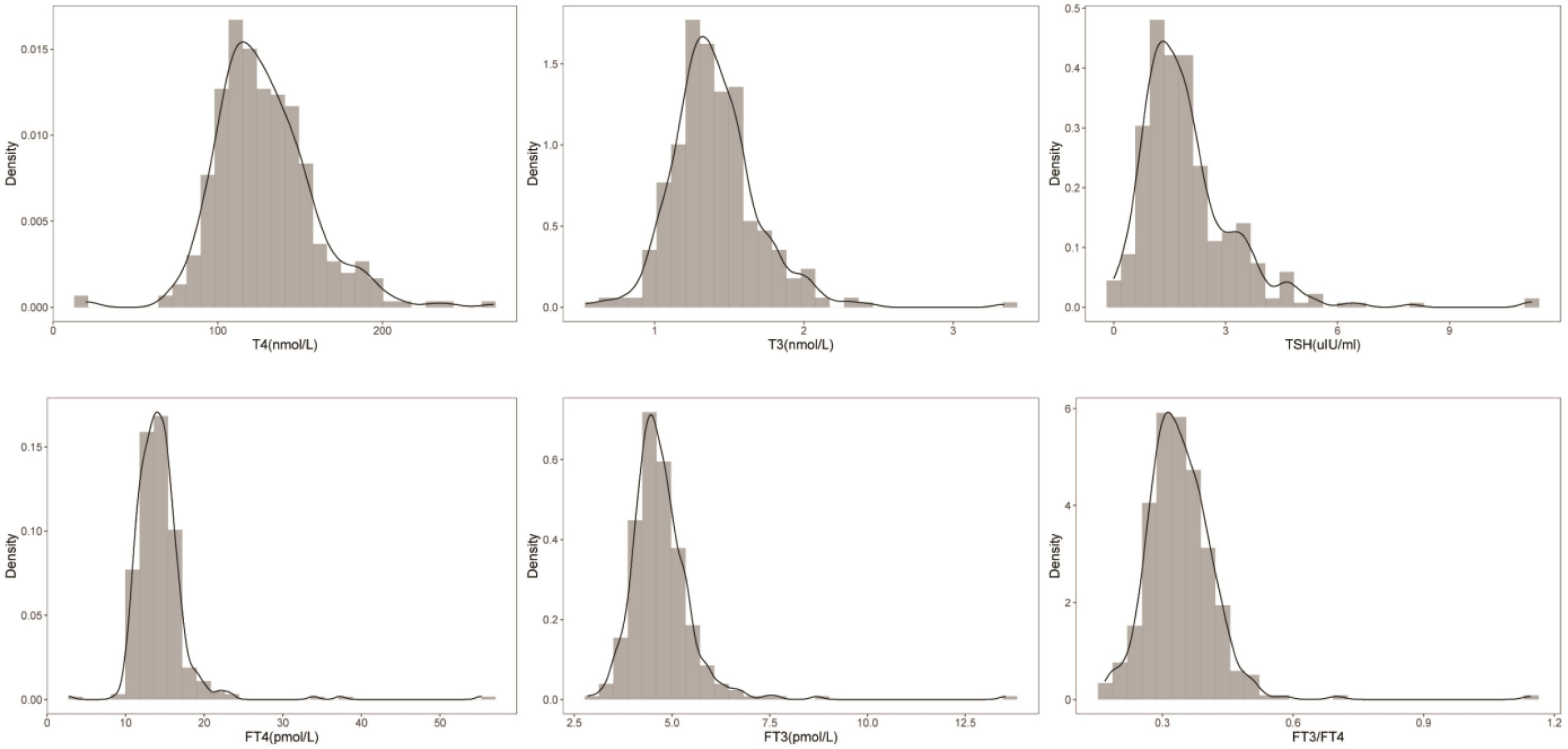
** Data distribution of FT4, T4, FT3, T3, TSH and FT3/FT4.** The normal distribution and density curves of the serum FT4, T4, FT3, T3, and TSH concentrations and the FT3/FT4 ratio were generated.

After screening the original data and eliminating samples with missing values, the current study included a total of 349 participants (139 males and 210 females). In line with the rigorous CLSI guidelines, the sample size of this study was set to include at least 120 patients for a 95th percentile clinical reference range determination with 95% confidence 15. Fig. 1 shows the distribution and density curves of the FT4, T4, FT3, T3, and TSH concentrations and the FT3/FT4 ratio. All these indices followed a normal distribution.

The main characteristics of the participants in this study, stratified by sex, are shown in Table 1. The average ages of the men and women in this study were 93.1 years and 93.5 years, respectively. There were few differences in body-related indices according to sex. Females had higher levels of both SBP and DBP than males. The ranges and the means of all the thyroid hormone levels are listed in the table.

### Correlations between thyroid hormone levels and body-related indices

First, we assessed the correlations between FT4, T4, FT3, T3, and TSH concentrations and between the FT3/FT4 ratio and the anthropometric parameters of interest. Correlations between hormone levels and sex were analyzed by the Spearman correlation coefficient, while other body-related indices were analyzed by the Pearson correlation coefficient. As presented in Table 2, the results revealed that sex was not correlated with the FT4, T4, FT3, T3, or TSH concentration or the FT3/FT4 ratio. Significant positive correlations were only observed between FT4 and age (r=0.208, p< 0.01), while other thyroid function test results were not sufficiently correlated with age from 90 to 108 years. Among the body-related indices, BMI and WHtR were correlated with some of the serum thyroid hormones levels. FT4 (r=-0.126, p< 0.05) was negatively correlated with BMI; while FT3 (r=0.149, *P*< 0.01), T3 (r=0.204, *P*< 0.01) and the FT3/FT4 ratio (r=0.202, *P*< 0.01) were strongly positive correlated with BMI. FT4 (r=-0.153, *P*< 0.01) was also negatively correlated with WHtR; FT3 (r=0.112, *P*< 0.05), T3 (r=0.162, *P*< 0.01) and the FT3/FT4 ratio (r=0.188, *P*< 0.01) were also positively related to the WHtR. T3 levels were significantly positive correlated with SBP (r=0.140, *P*< 0.01) and DBP (r=0.119, *P*< 0.05), while the FT3/FT4 ratio was positively correlated with SBP alone (r=0.105, *P*< 0.05).

**Figure 2:**
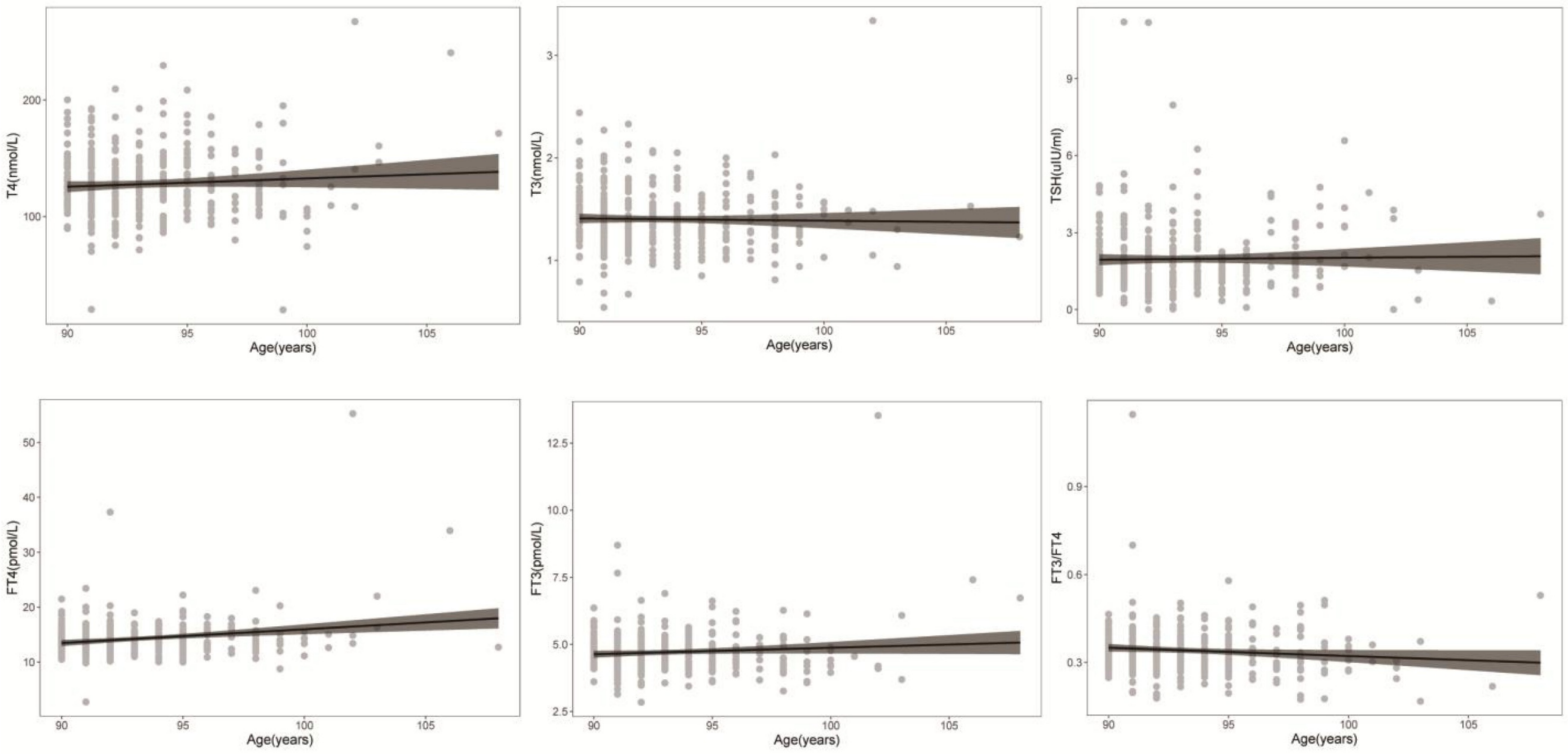
** Distribution of thyroid hormone levels with age.** Scatter plots of FT4, T4, FT3, T3, and TSH levels and the FT3/FT4 ratio with age. A regression curve was also plotted.

Scatterplots of FT4, T4, FT3, T3, and TSH concentrations and the FT3/FT4 ratio with age are shown in Fig. 2. Regression curves of thyroid hormone levels were also plotted. These figures reflect the variation tendency in different thyroid hormone levels from 90 to 108 years of age. FT4 increases with age, while FT3 does not change significantly with age; therefore, the FT3/FT4 ratio has a downward trend. T3, T4 and TSH did not significantly change with age among individuals over 90 years old.

### Outliers in thyroid function tests

On the basis of the reference intervals for common clinical biochemistry tests- Part 10 18: Serum triiodothyronine, thyroxine, free triiodothyronine, free thyroxine and thyroid stimulating hormone, the current Chinese standard RIs for adults are as follows: FT4 12.80-21.30 pmol/L, T4 70-140 nmol/L, FT3 3.85-6.30 pmol/L, T3 1.30-2.40 nmol/L, and TSH 0.75-5.60 μIU/ml. The numbers and percentages of patients with different thyroid function test results below, within and above the Chinese standard RIs are displayed in Table 3.

As much as 29.8% of the total subjects were considered to exceed the upper limit for T4. Moreover, up to 30.1% of the oldest-old people in this study were under the lower limit for FT4. The percentage of participants with T3 level within the upper and lower limits of Chinese standard RI was only 59.9%, and the percentage of participants with a lower T3 level was 39.5%. A total of 90.5% of the subjects had FT3 level within limits, and 88.8% of the subjects had TSH level within limits, which are lower than the requirements of the definition of the RI. Within the RI values, 95% of the values for apparently healthy individuals usually fall between the 2.5th and 97.5th percentiles of the distribution of test results of a reference (healthy) population.^15^

All these results indicate that the current Chinese standard RIs for adults are not suitable for long-living people in China. Under the current standard RI, an excess of healthy older adults may be diagnosed with thyroid-related diseases, which can lead to overdiagnosis and excessive medical treatment.

### Reference intervals of FT4, T4, FT3, T3, TSH and FT3/FT4 for the Chinese oldest-old

**Figure 3:**
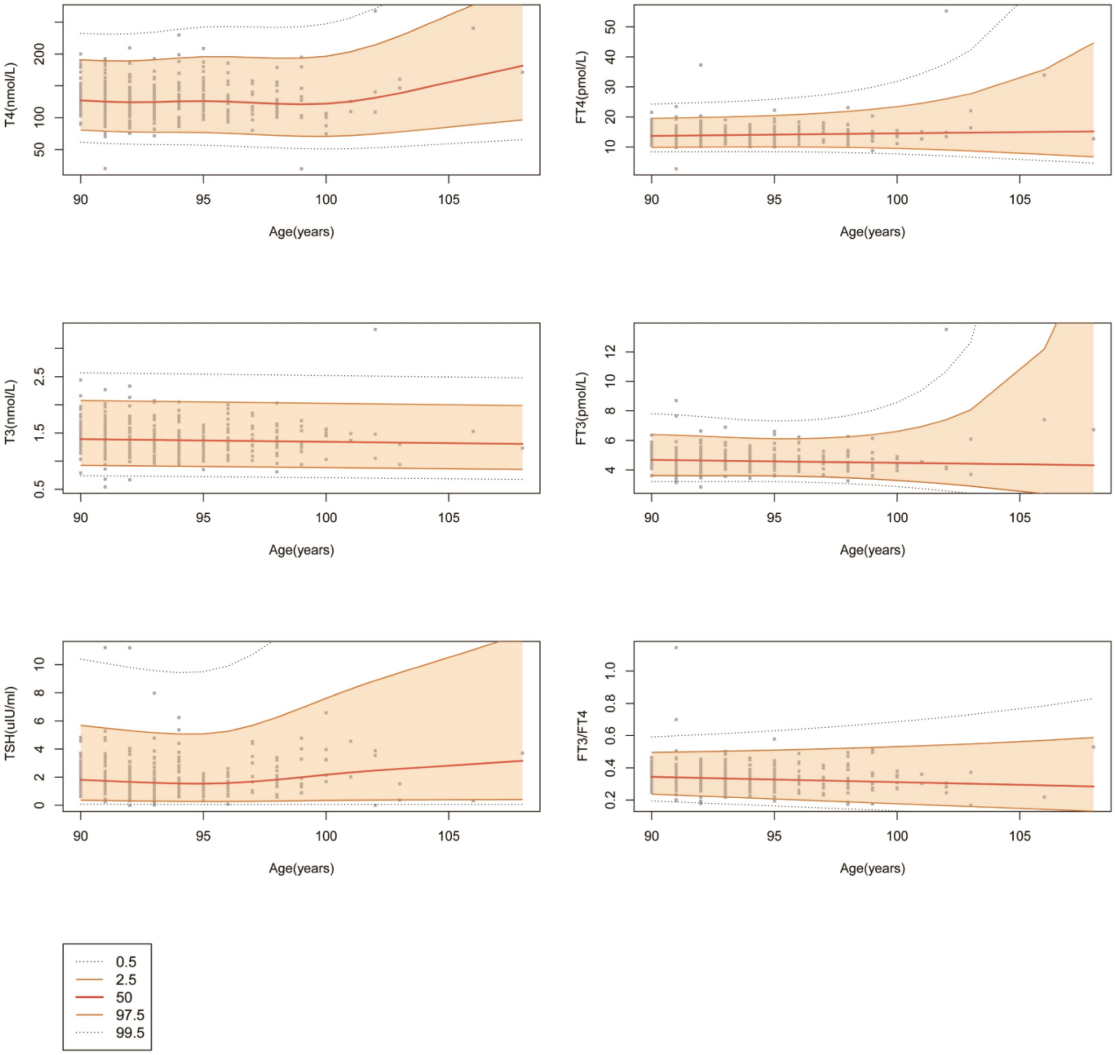
** The centile curves for FT4, T4, FT3, T3, TSH and FT3/FT4.** Changes in the median and 0.5, 2.5, 97.5, and 99.5 percentiles for thyroid-related hormones with age.

Using the Gamlss model, which is a generalized additive model for location scale and shape, we plotted the centile curves for the FT4, T4, FT3, T3, and TSH concentrations and the FT3/FT4 ratio. As shown in Fig. 3, 0.5, 2.5, 50, 97.5 and 99.5 percentile curves were drawn for the different thyroid biochemical test results. The thyroid hormone levels remained relatively stable between 90 and 100 years old. There is an escalating trend in median T4 level with age after 100 years, and the median TSH level began to increase after 95 years. The above results suggest that age should be considered when determining RIs for thyroid function test results.

Table 4 shows the 2.5, 5, 25, 50, 75, 95, and 97.5 percentage values for the FT4, T4, FT3, T3, and TSH concentrations and FT3/FT4 ratio in the oldest-old. The nonparametric method was used to establish the RIs for the oldest-old. According to the results described previously, there was no significant difference in thyroid hormone levels between men and women. Therefore, holistic RIs are constructed for the long-living population aged 90 years and older.

The RIs for the oldest-old were determined using the 2.5th percentile as the lower boundary and the 97.5th percentile as the upper boundary. All the RIs of the different thyroid hormones and TSH levels are shown in Table 5, together with the current Chinese standard RIs for adults. The desirable RIs of the serum thyroid hormone concentrations were as follows: T4 81-193 nmol/L; FT4 10.39-20.46 pmol/L; T3 0.94-2.05 nmol/L; FT3 3.56-6.43 pmol/L; TSH 0.29-5.28 μIU/ml; and FT3/FT4 ratio 0.197-0.496. Compared to the current standard, the RI suitable for long-lived seniors is considerably greater at T4. Furthermore, RIs for FT4 and T3 should be adjusted to a lower level than the current standard RIs. The RIs of FT3 levels in the oldest-old people have a wider range than those of their standard counterparts. Moreover, the lower limit of TSH is largely below the current RI, and the upper limit is also lower than the current limit. As a result, developing new RIs helps physicians better assess thyroid function in older adults and reduces the incidence of misdiagnosis of thyroid-related diseases.

In summary, the Chinese standard RIs of serum FT4, T4, FT3, T3, and TSH concentrations are not suitable for the oldest-old in China. Age needs to be considered when setting RIs. Based on the natural longevity cohort of Guangxi residents, we established the RIs of FT4, T4, FT3, T3, and TSH concentrations and the FT3/FT4 ratio for Chinese individuals older than 90 years.

## Discussion

As medical technology advances, both the global life expectancy and the percentage of elderly people in the total population are rising swiftly. The oldest-old, especially centenarians, is regarded as a sign of healthy aging in terms of avoiding life-threatening illness and maintaining normal functions 20. Studies based on the oldest-old can help us better investigate and demystify the mechanisms of healthy aging, thereby alleviating the medical and economic challenges induced by the aging population. Considering the increasing number of the oldest-old, standardized diagnoses and treatments for the elderly thyroid diseases need to be established to improve clinical management and promote healthy aging. Thyroid function varies between the oldest-old individuals and younger adults because of physiological changes in the HPT axis, the presence of geriatric syndromes, and the decline in various organ functions 11. There is a lack of appropriate RIs for the serum FT4, T4, FT3, T3, and TSH concentrations for the oldest-old over 90 years old. The impact of thyroid hormone levels on lifespan in the healthy oldest-old, which differs from that in the rest of the population, needs to be further investigated.

In the present study, we analyzed thyroid-related hormone levels in people older than 90 years in Guangxi, China, and explored the relationships of these hormone levels with body-related indices and blood pressure. In addition, for the first time, we established appropriate RIs based on CLSI guidelines for the oldest-old in China and compared them to current standards.

Studies conducted in the past have demonstrated a connection between thyroid hormone levels and BMI in adults from different countries. The study by Sakurai *et al.*, which included 1,044 men and 993 women in Japan, revealed a strong positive link between serum TSH levels and BMI in male subjects 21. In the USA, Kitahara *et al.* analyzed 1,623 men and 1491 women and suggested that TSH and FT3 were positively associated with BMI, while no associations were found between FT4 and BMI 22. A large-scale Chinese population-based study including 13,855 individuals reported a positive relationship between TSH level and BMI in females 23. In contrast, a study conducted by Manji *et al.* in the United Kingdom did not find a correlation between either serum TSH or FT4 levels and BMI in euthyroid individuals 24.

In this study, we found that FT4, FT3, and T3 concentrations and the FT3/FT4 ratio were strongly correlated with BMI in the Chinese oldest-old. The nutritional status of the oldest-old differs from that of the younger population, resulting in wide variance in BMIs. The average BMI in China is 24.1 kg/m^2^ according to a survey of 15,770,094 eligible participants with a median age of 40 years and a 52.8% proportion of males 25. In this study, the average BMIs of female and male subjects older than 90 years were 20.78 kg/m^2^ and 21.60 kg/m^2^, respectively, which are lower than the BMIs in the general adult population. Differences in dietary structure and glucose, lipid, and protein metabolism may be the contributing factors to the relationship between BMI and thyroid hormone levels.

In addition, we found significant correlations of FT4, FT3, and T3 levels and the FT3/FT4 ratio with the WHtR in the Chinese oldest-old. Consistent with our results, Ma D *et al.* also reported a significant and positive association between BMI and WHtR in their cross-sectional study of overweight adults in China 26. Another report based on obese children without a history of thyroid pathology in China revealed that the WHR was associated with TSH and the FT3/FT4 ratio 27. Furthermore, a cross-sectional survey of 2,483 subjects in China reported a negative correlation between waist circumference and TSH levels 28. As a result, there is an interaction between thyroid hormone levels and body thinness or fat distribution.

The serum TSH concentration is one of the most sensitive indicators of human thyroid status. However, there are very limited studies on TSH levels in populations older than 90 years. Although we did not find a significant association between TSH and age in the population older than 90 years, we observed that the Chinese oldest-old had 2.5 and 97.5 centile limits of serum TSH concentration lower than the current standard RI for adults.

Domestic and international studies have also shown that aging impacts the serum TSH reference range, but the causal relationships and mechanisms have not yet been determined. The National Health and Nutrition Examinations Survey III (NHANES III) in the United States reported a study involving adults with normal thyroid function that showed that for every 10 years of age increase over 40 years, the upper limits of the serum TSH concentration increased gradually 29. Another study based on a Chinese population reported similar results. After analyzing 7,693 individuals from different age groups, Chen J *et al.* reported that the 97.5th percentile increased in individuals older than 40 years, and the upper limit of individuals older than 70 years was 8.07 mIU/L 9. Studies have demonstrated that the median and reference intervals of serum TSH are greater in older adults than in younger ones, with a trend of increasing values as age progresses 30, 31. A cross-sectional survey revealed that the serum TSH level was significantly higher in the oldest-old people aged 98 years old than in another population with an average age of 72 years 32.

However, Diana van Heemst concluded that the majority of adults older than 65 years have serum TSH concentrations within the reference range 6. Other study results also indicated high stability of serum TSH concentrations in most people over time 33. Tozzoli *et al.* analyzed individuals from northern Italy and reported that the median TSH concentration progressively decreased from 0-4 to 85-104 years in the overall population, but no correlation was found between the serum TSH concentration and age 34. One study showed that serum TSH concentrations in centenarians are lower than those in elderly and younger individuals aged 65-80 years and 20-64 years, respectively 35. This inconsistency may be attributed to differences in race, sampling and sample sizes of the population. Besides, differences in community-based or hospital-based surveys may lead to bias in results. More importantly, cross-sectional studies provide information only on distributions and not evidence of causation. Therefore, additional population studies and cohort evidence based on the oldest-old individuals, especially centenarians, are needed to further elucidate the associations of TSH with age and longevity.

Differences in thyroid hormone levels with age have also been observed and analyzed in previous studies, but no consistent conclusions have been reached. After analyzing specimens from 2,380 apparently healthy individuals from different cities in China, Wang D *et al.* reported a significant negative relationship between age and FT3 in males 36. Another study with 6,781 subjects included from hospitals in China revealed no differences in TSH or FT4 levels according to age, sex or overall, but FT3 levels significantly decreased with age 37. Ma C *et al.* reported that both FT3 and FT4 decreased with increasing age and reported differences in RIs for serum FT3, FT4 and TSH levels compared with standard RIs 38. Similar findings reported by Mariotti, S *et al.* suggested that serum FT3 concentrations in centenarians are lower than those in individuals aged 65-80 years and 20-64 years 35. Wang Y and colleagues showed that FT4, FT3, and total T3 levels in males and FT4 levels in females were inversely correlated with age. Significant differences in thyroid hormone levels were also found between patients younger and older than 50 years old 39. A cohort study with a 6.5-year interval showed that FT4 levels increase over time, with a more pronounced increase in elderly individuals 40. The American Cardiovascular Research Survey conducted a study including 843 subjects with an average age of 72 years and revealed increases in TSH and FT4 levels as well as a decrease in T4 within 13 years of follow-up 41. An expert consensus in China proposed that the HPT axis changes with age in healthy elderly people, as indicated by decreased T3 and FT3 levels, slightly increased or retained FT4 levels, a decreased FT3/FT4 ratio, and increased TSH levels 11.

On the basis of the present study, our results suggested that the serum FT4 concentration is correlated with age in the oldest-old. Both the lower and upper limits of RIs for FT4 and T3 are below the counterpart values for general adults in China, while the RI of T4 was greater in the oldest-old than in the general population. Furthermore, we found that the oldest-old have a wider range of FT3 RIs than younger adults. Our findings complement the deficiency of thyroid hormone levels in the population older than 90 years and provide a new rationale for improving thyroid function in the oldest-old.

The age-specific TSH reference range may minimize the underdiagnosis of subclinical hypothyroidism in young adults and prevent overdiagnosis in elderly individuals 42. Our results suggest that using age-specific RIs can reduce the rate of abnormalities in thyroid function tests. When the Chinese standard RIs of T3, T4 and FT4 were applied in the healthy oldest-old, the proportions were only 59.9%, 69.6% and 67.6%, respectively. On the other hand, using our TSH RI instead of the current RI to assist in the diagnosis of the elderly thyroid diseases may reduce misdiagnosis by 6.2%. Therefore, it is inappropriate to use the current RIs to determine the thyroid status of the oldest-old.

In coherence with our results, a Chinese population-based study showed that Adults over 65 exhibited a significantly greater prevalence of subclinical and clinical hypothyroidism than younger adults, according to the general population's reference range. The prevalence of subclinical hypothyroidism was reduced from 19.87% to 3.3% and the prevalence of clinical hypothyroidism was reduced from 2.09% to 1.6%, in elderly individuals older than 65 years via the application of age-specific RIs 30. Experts have noted that elevated TSH levels and altered thyroid hormones should not be considered the only indicators of the elderly thyroid diseases; a more comprehensive assessment is needed instead 43. Therefore, caution should be exercised when diagnosing thyroid diseases and disorders in elderly individuals as well as when determining the need for thyroid hormone supplementation.

The age-related changes in the HPT axis may be a protective mechanism due to worsening metabolism in elderly individuals, reflected in reduced T4-to-T3 conversion, weakened inhibition of TSH feedback, and increased TSH levels. Moreover, an increase in TSH might also be associated with an increase in the tuning of the thyroid hormone response to TSH or a decrease in TSH biological activity with aging 11. Chronic disease prevalence and medication usage among older individuals make comparisons with young people more complicated and confounding. Studies have suggested that chronic disease is associated with reduced concentrations of T3, TSH and FT4, which may also be influenced by disease severity and duration 6. In addition, genetics, age, ethnicity, sex, and environmental factors, including iodine status, smoking status, circadian rhythmicity, season and temperature, also contribute to variations in thyroid status, resulting in different research conclusions 40, 44, 45.

There are some limitations to this survey: 1) Although participants were rigorously screened as 'apparently healthy' based on self-reported medical history and exclusion criteria, undiagnosed subclinical conditions (e.g., cardiovascular diseases or diabetes) or medications (e.g., non-thyroid drugs with indirect hormonal effects) might still confound the results. 2) The study focused on a longevity population sample from a specific area in Guangxi Province, and findings related to geographical, ethnic, and genetic factors should be confirmed and expanded in a broader national longevity population in future research. 3) Since this was a cross-sectional study, the findings cannot establish causal relationships.

Despite the existence of inconsistencies, our results provide new insights and evidence on trends in thyroid hormone levels with age in the Chinese oldest-old. We will focus on the longevity cohort in a follow-up study to explore the role of thyroid hormones in longevity and investigate causal associations further.

## Figures and Tables

**Table 1:** Baseline Characteristics.

Characteristic	Female		Male	
	Range	Mean ± SD	Range	Mean ± SD
Age (years)	90-106	93.5±3.0	90-108	93.1±3.0
BMI (kg/m^2^)	10.54-42.54	20.78±4.68	12.11-41.47	21.60±4.02
WHR	0.77-1.16	0.938±0.062	0.78-1.17	0.936±0.060
WHtR	0.43-0.93	0.5757±0.0729	0.39-0.67	0.5361±0.0578
SBP (mmHg)	92-217	159.2±25.9	88-225	149.3±25.2
DBP (mmHg)	50-129	84.4±13.4	47-149	81.1±13.6
T4 (nmol/L)	19.9-267.3	130.46±29.4	70.1-229.6	124.97±28.27
FT4 (pmol/L)	8.76-55.25	14.386±4.221	10.19-23.07	14.296±2.249
T3 (nmol/L)	0.68-3.34	1.432±0.313	0.67-2.16	1.367±0.246
FT3 (pmol/L)	3.33-13.53	4.725±0.947	2.84-6.73	4.707±0.634
TSH (μIU/ml)	0-11.19	1.854±1.273	0.45-11.21	2.158±1.451
FT3/FT4	0.17-0.70	0.339±0.070	0.17-0.58	0.338±0.070

**Table 2:** Correlations of serum FT4, T4, FT3, T3, and TSH concentrations and the FT3/FT4 ratio with clinical variables.

		T4	FT4	T3	FT3	TSH	FT3/FT4
Sex	ρ	-0.102	0.058	-0.069	0.069	0.104	0.002
	*P* value	0.058	0.281	0.201	0.201	0.053	0.971
Age	r	0.070	0.208**	-0.023	0.085	0.016	-0.104
	*P* value	0.191	0.000	0.663	0.111	0.771	0.052
BMI	r	-0.073	-0.126*	0.204**	0.149**	-0.021	0.202**
	*P* value	0.177	0.019	0.000	0.005	0.701	0.000
WHR	r	-0.037	-0.084	-0.067	-0.037	0.100	0.027
	*P* value	0.495	0.119	0.210	0.490	0.063	0.613
WHtR	r	-0.004	-0.153**	0.162**	0.112*	-0.078	0.188**
	*P* value	0.934	0.004	0.002	0.036	0.145	0.000
SBP	r	0.027	-0.064	0.140**	0.073	0.019	0.105*
	*P* value	0.619	0.234	0.009	0.177	0.719	0.050
DBP	r	0.056	-0.036	0.119*	0.082	-0.037	0.089
	*P* value	0.297	0.499	0.026	0.127	0.496	0.096

ρ, Spearman correlation coefficient r, Pearson correlation coefficient ^*^, two-tailed significance < 0.05 ^**^, two-tailed significance < 0.01

**Table 3:** Numbers of outliers in different tests compared to Chinese standard RIs

	RIs	Under		Within		Exceed	
		number	%	number	%	number	%
T4 (nmol/L)	70-140	2	0.6	243	69.6	104	29.8
FT4 (pmol/L)	12.80-21.30	105	30.1	236	67.6	8	2.3
T3 (nmol/L)	1.30-2.40	138	39.5	209	59.9	2	0.6
FT3 (pmol/L)	3.85-6.30	23	6.6	316	90.5	10	2.9
TSH (μIU/ml)	0.75-5.60	33	9.5	310	88.8	6	1.7

**Table 4:** The percentiles for serum FT4, T4, FT3, T3, and TSH concentrations and the FT3/FT4 ratio.

Percentiles	T4 (nmol/L)	FT4 (pmol/L)	T3 (nmol/L)	FT3 (pmol/L)	TSH (μIU/ml)	FT3/FT4
2.5	81.3450	10.39150	0.94000	3.55850	0.29250	0.1966
5	89.5500	10.83100	1.00700	3.68400	0.49000	0.2328
25	108.9500	12.37000	1.23000	4.27500	1.12000	0.2936
50	125.2000	13.94000	1.37000	4.60000	1.68000	0.3329
75	144.4500	15.42000	1.55000	5.03000	2.44000	0.3827
95	184.2900	18.73100	1.93900	5.89900	4.53900	0.4543
97.5	192.9600	20.46150	2.05000	6.43300	5.28150	0.4956

**Table 5:** RIs for Chinese adults and the oldest-old.

	Oldest-old RIs		Current RIs	
	Lower limit	Upper limit	Lower limit	Upper limit
T4 (nmol/L)	81	193	70	140
FT4 (pmol/L)	10.39	20.46	12.80	21.30
T3 (nmol/L)	0.94	2.05	1.30	2.40
FT3 (pmol/L)	3.56	6.43	3.85	6.30
TSH (μIU/ml)	0.29	5.28	0.75	5.60

## Abbreviations

T4: Thyroxine
T3: Triiodothyronine
FT4: Free thyroxine
FT3: Free triiodothyronine
TSH: Thyroid-stimulating hormone
HPT: Hypothalamic-pituitary-thyroid
TBG: Thyroxine-binding globulin
TBPA: Thyroxine-binding prealbumin
RI: Reference interval
CLSI: Clinical and Laboratory Standards Institute
WHR: Waist hip ratio
WHtR: Waist height ratio
SBP: Systolic blood pressure
DBP: Diastolic blood pressure
EDTA: Edetate
SD: Standard deviation
BMI: Body mass index
